# Negative workplace gossip and organizational citizenship behavior in the Chinese kindergarten teaching workforce: exploring interpersonal trust as a mediator

**DOI:** 10.3389/fpsyg.2026.1794504

**Published:** 2026-03-20

**Authors:** Lili Xu, Baoan Feng, Shengli Mao

**Affiliations:** College of Teacher Education, Quzhou University, Zhejiang, China

**Keywords:** Chinese sample, interpersonal trust, kindergarten teachers, negative workplace gossip, organizational citizenship behavior

## Abstract

**Introduction:**

While the empirical connection between negative workplace gossip and organizational citizenship behavior has been extensively documented, the psychological mechanisms underpinning this relationship remain insufficiently explored, particularly among Chinese kindergarten teachers. Based on social exchange theory, this study examines the association between negative workplace gossip and organizational citizenship behavior among Chinese kindergarten teachers, along with the mediating effect of interpersonal trust in this pathway.

**Methods:**

This cross-sectional survey employed a convenience sampling method to collect self-reported data on negative workplace gossip, organizational citizenship behavior, and interpersonal trust from 1,423 kindergarten teachers in China. Statistical analyses were carried out using Model 4 of the PROCESS macro (Version 4.1) in SPSS 29.0, with indirect effects evaluated by means of bias-corrected bootstrapping (5,000 resamples).

**Results:**

Results reveal that negative workplace gossip is negatively associated with organizational citizenship behavior, and interpersonal trust partially mediates this pathway.

**Discussion:**

This study extends the application of social exchange theory to the field of early childhood education, enriches the academic literature on workplace gossip and organizational citizenship behavior, and offers theoretical implications for promoting positive workplace behaviors among Chinese kindergarten teachers.

## Introduction

The occupational wellbeing and interpersonal workplace environment of kindergarten teachers have become increasingly important for the stable and high-quality development of early childhood education in China. As a key indicator of teachers' proactive and prosocial functioning, organizational citizenship behavior describes educators' voluntary, non-mandatory contributions beyond formal role expectations, which is positively associated with improvements in school climate and educational quality (Bogler and Somech, 2023; Triningsih et al., 2023; Gnanarajan et al., 2022). Organizational citizenship behavior refers to employees' discretionary, extra-role conduct that falls outside formal job descriptions and is not directly rewarded by organizational incentives, yet collectively boosts operational effectiveness and long-term organizational resilience (Organ, 1988). These behaviors span prosocial, constructive actions, such as assisting colleagues with work challenges, engaging proactively in organizational initiatives, and maintaining a positive mindset during professional obstacles. Organizational citizenship behavior is positively associated with organizational success and is linked to higher operational efficiency—predicting performance outcomes and being associated with stronger adaptive capacity to uncertainty (Han et al., 2022; Santa et al., 2023)—and is related to better internal brand cultivation, particularly in service sectors like hospitality and healthcare, where it is associated with brand promise delivery and sustainable brand growth (Abdel Majeed et al., 2024; Liao et al., 2025).

Beyond organizational benefits, organizational citizenship behavior fosters employee wellbeing by creating a virtuous cycle between prosocial actions and multifaceted happiness, strengthening employees' sense of belonging and loyalty (Unanue et al., 2021). Additionally, organizational citizenship behavior is positively associated with better team collaboration and interpersonal dynamics and is linked to greater information sharing, being associated with lower levels of conflicts, and being associated with higher levels of employees' social network centrality, while also being related to higher levels of interpersonal intelligence that support more sustainable prosocial behaviors (Han et al., 2022). Additionally, organizational citizenship behavior enhances team collaboration and interpersonal dynamics by promoting information sharing, reducing conflicts, and increasing employees' social network centrality, while also nurturing interpersonal intelligence for more sustainable prosocial behaviors (Han et al., 2022). Given the substantial significance of organizational citizenship behavior for organizational effectiveness, prior research has explored its antecedents and confirmed that workplace deviant behaviors (e.g., incivility, interpersonal conflict, workplace ostracism) relate to employees' engagement in organizational citizenship behavior (Annalakshmi et al., 2022; Gümüştaş and Karataş Gümüştaş, 2023; Tran, 2025). A review of existing literature reveals that scholarship examining the connection between negative workplace gossip and organizational citizenship behavior remains limited, with available studies restricted to specific occupational groups such as frontline staff and their direct supervisors (Xie et al., 2019). Comprehensive investigations within educational contexts are still absent.

Among educators, organizational citizenship behavior—discretionary, prosocial contributions that extend beyond formal role obligations to benefit peers and educational organizations—encompasses both individual-oriented actions (e.g., offering support to fellow teachers) and institution-oriented actions (e.g., upholding organizational values), and acts as a pivotal catalyst for educator performance and institutional effectiveness (Hermanto and Srimulyani, 2022; Rahman and Karim, 2022). Validated as a cross-contextual construct spanning secondary schools, vocational training colleges, and private higher education institutions globally, educators' organizational citizenship behavior serves as an essential foundation for fostering collaborative educational environments and achieving institutional development objectives (Hermanto and Srimulyani, 2022; Shaya et al., 2024). Multiple studies have demonstrated that organizational citizenship behavior is positively associated with organizational justice, ethical and transformational leadership practices, effective interpersonal communication, and individual personality traits, with work engagement, job satisfaction, and quality of work life showing an associative role (Hermanto et al., 2024; Hermawan and Susanti, 2022; Phetsombat and Na-Nan, 2023).

Negative workplace gossip is conceptualized as informal, derogatory, and largely unsubstantiated discourse about colleagues within organizational contexts, which generally takes place in the absence of the targeted individual (Kakarika et al., 2024; Xie et al., 2024). Distinguished from overt workplace deviant behaviors—such as bullying (direct, repeated harm), ostracism (intentional exclusion), and incivility (low-intensity discourtesy)—negative workplace gossip is covert, socially contagious, and insidious, and is associated with eroded reputations and infiltrated team networks with lingering effects (Cheng X. et al., 2023). Although prior research notes its marginally constructive functions (e.g., monitoring unethical conduct; Gabriel et al., 2024), its detrimental impacts far outweigh these benefits and deserve greater scholarly attention. At the individual level, exposure to negative workplace gossip is negatively associated with job satisfaction, subjective wellbeing, and in-role performance, while being positively associated with turnover intentions and negatively associated with proactive behaviors like knowledge sharing—actions analogous to organizational citizenship behavior (Cheng B. et al., 2023; Gao et al., 2024). From an organizational perspective, it is negatively associated with interpersonal trust, team dynamics, and collective organizational performance (Xie et al., 2022).

Despite growing evidence of negative workplace gossip's harms, its specific link with organizational citizenship behavior remains underexplored, particularly among Chinese kindergarten teachers. In China, kindergarten teachers, who hold recognized professional qualifications in the field of early childhood education, are tasked with designing and implementing structured learning experiences as well as providing developmental care to children aged 3–6 years old (with regional variations in adherence to national regulatory guidelines) within regulated early childhood education institutions (Feng et al., 2025). This professional group operates within a distinct occupational landscape, and these unique characteristics not only define the parameters of their roles but may also be associated with a higher prevalence of detrimental workplace gossip and a negative association between such gossip and the collaborative dynamics required to deliver high-quality services (Feng et al., 2025). Globally, women make up over 90% of kindergarten teachers (Hong et al., 2022; Piperac et al., 2024), a demographic associated with stereotypes of a propensity for gossip (Robbins and Karan, 2020). Research notes women often use relational aggression (e.g., gossip) against female peers (Crothers et al., 2009; Namie, 2021). Combined with the role's heavy emotional labor demands (Sottimano et al., 2017), negative gossip is negatively linked to trust, is associated with emotional exhaustion (Itzkovich and Dolev, 2021), and shows a negative relationship with organizational citizenship behavior—critical for team harmony and quality education.

While emerging evidence links negative workplace gossip to organizational citizenship behavior, this association is likely indirect, operating through third-party mechanisms—with interpersonal trust as a pivotal mediator. Interpersonal trust is defined as an individual's optimistic expectation that other parties will prioritize their interests, honor their commitments, and avoid harmful actions (Rousseau et al., 1998). Within organizational contexts, interpersonal trust acts as a cornerstone of individual adaptation, wellbeing, and professional growth, while also showing a positive correlation with collaborative team dynamics, being positively associated with group creativity and decision-making quality, and linking to positive organizational attitudes and overall effectiveness (Dirks and de Jong, 2022; Hancock et al., 2023; Raatikainen et al., 2023). To our knowledge, no prior investigation has explored interpersonal trust as a mediating variable in the relationship between negative workplace gossip and organizational citizenship behavior, especially within the cultural context of Chinese educational institutions. Existing studies demonstrate that negative workplace gossip is negatively associated with colleague friendships and shows a relationship with interpersonal conflicts (Liu, 2024), which in turn are linked to lower levels of mutual trust among colleagues. Drawing on social exchange theory (Cropanzano and Mitchell, 2005), reciprocal workplace cooperation is built on a foundation of trust. When trust is undermined, the bedrock of social exchange collapses, and employees' willingness to engage in organizational citizenship behavior—voluntary extra-role actions benefiting the organization—is associated with a reduction or complete absence (Aryee et al., 2002; Wat and Shaffer, 2005). This study therefore focuses on Chinese kindergarten teachers to examine the influence of negative workplace gossip on organizational citizenship behavior and the mediating role of interpersonal trust. The findings aim to provide a theoretical basis and practical implications for facilitating positive workplace behaviors among kindergarten teachers.

### Theoretical background and hypotheses

The present investigation draws on social exchange theory as its core analytical framework to examine the association between negative workplace gossip and organizational citizenship behavior among Chinese kindergarten teachers. Social exchange theory centers on the voluntary interactions among individuals driven by the expectation and actual attainment of mutual returns (Blau, 1964; Cropanzano and Mitchell, 2005). Specifically, it explains how social relationships are constructed and maintained through the reciprocal exchange of resources or favors, which constitute the concrete manifestations of social exchange. These exchanges encompass both material (e.g., economic support) and non-material (e.g., emotional care, information sharing, mutual daily assistance) interactions, and are not rigidly contractual but involve diffuse future obligations rather than precisely specified terms. Three core principles underpin social exchange and lay the foundation for understanding workplace interactions. First, voluntary: Actions coerced by physical force are excluded from the scope of social exchange, while compliance with other forms of power may be deemed voluntary if motivated by the expectation of obtaining corresponding benefits (Blau, 1964). Second, reciprocity: As the foundational principle of the theory, the need to repay received benefits serves as a core driver of social interaction, fostering the formation of social networks and group structures; over time, this inherent need is correlated with widely recognized group norms of reciprocity (Cooney, 2022; Ahmad et al., 2023). Third, trust: Establishing and sustaining exchange relations requires individuals to make commitments to each other and trust in others' willingness to reciprocate (Lengieza et al., 2023). Such trust shows a positive relationship with consistent fulfillment of obligations and long-term investment in maintaining relationships (Tarkang Mary and Ozturen, 2019). Rooted in these principles—particularly reciprocity and trust, the two most critical tenets for interpersonal exchange dynamics—social exchange theory posits that workplace interactions are governed by implicit norms of mutual obligation. For employees, engaging in prosocial behaviors (e.g., organizational citizenship behavior) is a voluntary act driven by the expectation of reciprocal respect, support, or other non-material resources from colleagues and the organization (Blau, 1964; Cropanzano and Mitchell, 2005). Notably, this study focuses on interpersonal trust (rather than organizational trust) because social exchange theory emphasizes that micro-level interpersonal exchanges among colleagues are the foundation of broader workplace exchange relationships. Negative workplace gossip, as a destructive interpersonal behavior, directly violates the reciprocity norm of social exchange theory by disseminating derogatory, unsubstantiated information about colleagues—being negatively linked to the mutual respect and goodwill required for reciprocal exchange. This violation, in turn, is associated with lower levels of interpersonal trust between colleagues, as gossip signals that the gossipers (and potentially the broader peer group) are unwilling to uphold the reciprocity and goodwill that underpin trust. By explicitly grounding the analysis in these social exchange theory tenets, this study investigates the link between negative workplace gossip and organizational citizenship behavior in the Chinese kindergarten teaching workforce, as well as the mediating role that interpersonal trust (a core micro-level exchange resource) plays in this relationship.

### Negative workplace gossip and organizational citizenship behavior

Negative workplace gossip, as a widespread form of social sabotage, is associated with a chain of harmful psychological and relational outcomes that show a negative correlation with employees' willingness to participate in voluntary, organization-benefiting actions (Agina et al., 2023; Aboramadan et al., 2021). By disseminating derogatory and unverified information, such gossip is linked to a hostile social climate that signals a lack of respect and safety (Touni and Hussien, 2023), and shows an association with threats to employees' psychological resources and relational attachments (He and Wei, 2022; Jung and Yoon, 2022). Targeted individuals often experience heightened emotional distress, diminished self-worth, and a sense of social exclusion (Shi et al., 2022), which are negatively related to the cognitive and motivational energy required for discretionary prosocial behavior (Hase et al., 2021; Zimmerman et al., 2016). For kindergarten teachers, whose work is inherently relational and dependent on mutual trust to ensure effective collaboration and child care (Feng et al., 2025), these negative effects are amplified—gossip shows a negative relationship with the interpersonal harmony that underpins their daily work, and is correlated with cynicism toward colleagues and the organization. Drawing on both social information processing theory (Salancik and Pfeffer, 1978) and social exchange theory, negative workplace gossip acts as a harmful social signal that frames employees' perceptions of the interpersonal exchange environment (rather than just the organizational environment) as untrustworthy and unsupportive (Baer et al., 2018). From a social exchange theory perspective, this signal is linked to violations of the reciprocity norm: gossipers fail to provide the respect and goodwill that colleagues expect in reciprocal exchange, and this failure is associated with targeted employees' withdrawal from voluntary prosocial contributions (e.g., organizational citizenship behavior) as a way to avoid further imbalance in the exchange relationship. When teachers interpret such gossip as a sign of relational breakdown, their organizational identification and sense of belonging weaken, and they are less inclined to invest extra effort in organizational citizenship behavior—actions that rely on a foundation of reciprocal trust and commitment to collective wellbeing. Empirical evidence supports this logic: studies in educational settings have linked interpersonal mistreatment, including gossip, to reduced helping, courtesy, and civic virtue among teaching staff (Akkaya, 2019; Kaya, 2015). Cross-occupational research also confirms that gossip-induced resource depletion shows an association with withdrawal from extra-role contributions (Zeng et al., 2022). Given the high interdependence of kindergarten teaching and the essential function of organizational citizenship behavior in sustaining team performance and care standards, negative workplace gossip is hypothesized to be negatively associated with organizational citizenship behavior.

*Hypothesis 1: Negative workplace gossip is inversely related to organizational citizenship behavior among Chinese kindergarten teachers*.

### Interpersonal trust as a mediating factor

As a core underpinning of positive workplace interactions and social exchange, interpersonal trust encompasses three core dimensions: the belief in others' competence, the expectation of their benevolence, and the confidence in their behavioral reliability (Abrams et al., 2003; Calhoun et al., 2019). It is identified as a pivotal psychological foundationassociated with sustaining collegial collaboration, showing a positive correlation with resource sharing, and linking to emotional connections among organizational members (Cropanzano and Mitchell, 2005). A critical function of interpersonal trust is its association with reduced uncertainty and risk perceptions in interpersonal dynamics, and it shows a relationship with psychological assurance for voluntary cooperation and discretionary extra-role contributions. For kindergarten teachers in particular, stable interpersonal trust is characterized as an indispensable prerequisite linked to seamless coordination in childcare services and positively associated with overall team effectiveness (Chang et al., 2025). From the perspective of social exchange theory, interpersonal trust is not only a core principle of social exchange but also a critical “exchange resource” that enables reciprocal interactions. When colleagues uphold the norm of reciprocity (e.g., treating each other with respect, providing support), interpersonal trust shows higher levels; conversely, behaviors that violate reciprocity (e.g., negative workplace gossip) are negatively linked to this trust. However, employees exposed to negative workplace gossip often experience significant erosion of their interpersonal trust (Pápay, 2021; Estévez, 2021): the derogatory information disseminated through gossip is associated with violations of social exchange theory's reciprocity norm by showing a negative association with the mutual respect and goodwill that colleagues owe each other in social exchange, which in turn is correlated with lower levels of their perceptions of colleagues' benevolence and reliability, shows a negative relationship with the reciprocal norms and collaborative foundations among peers (Guo et al., 2021; D'Cruz and Noronha, 2012), and consequently inhibits their willingness to make proactive contributions to the organization. Empirical evidence has corroborated that negative workplace interactions are negatively related to employees' prosocial behavioral tendencies and show an association with eroded interpersonal trust (Xie et al., 2022). For kindergarten teachers, whose work relies heavily on intensive team collaboration and cross-role coordination, the depletion of interpersonal trust is negatively associated with the collaborative climate essential to childcare delivery, and this depletion is linked to teachers' reluctance to invest extra effort in organizational citizenship behavior.

This underlying mechanism can be thoroughly explained using social exchange theory (Blau, 1964; Cropanzano and Mitchell, 2005). From this theoretical standpoint, organizational citizenship behavior is fundamentally an outcome of trust-founded social exchange, whose occurrence depends on employees' beliefs that voluntary contributions are linked to balanced reciprocal rewards. As a destructive form of negative interpersonal interaction, negative workplace gossip is systematically negatively related to this exchange dynamic by being associated with violations of the reciprocity norm of social exchange theory—gossipers fail to reciprocate the respect and goodwill that colleagues extend, and this failure is correlated with reduced teachers' confidence in colleagues' core trustworthiness dimensions (e.g., competence, benevolence, reliability). Importantly, this erosion targets interpersonal trust (between colleagues) rather than organizational trust (in the institution), as gossip is a micro-level interpersonal behavior that shows a negative relationship with peer-to-peer exchange relationships. For kindergarten teachers, whose childcare work depends on intensive cross-role collaboration, this trust depletion is linked to higher perceived risks of reciprocity failure, and this depletion is associated with teachers' reluctance to invest in voluntary organizational citizenship behavior (Coyle-Shapiro and Kessler, 2000; Dwiyanti and Hidayah, 2022). Conversely, employees with robust interpersonal trust—rooted in beliefs in colleagues' competence, benevolence, and reliability—are more likely to engage in organizational citizenship behavior, as they anticipate positive responses to their discretionary efforts within the scope of social exchange theory (Blau, 1964; Cropanzano and Mitchell, 2005).

*Hypothesis 2: Interpersonal trust mediates the negative relationship between negative workplace gossip and organizational citizenship behavior among Chinese kindergarten teachers*.

**Figure 1:**
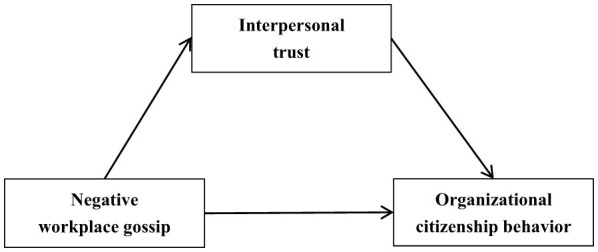
Mediating framework.

Grounded in social exchange theory (Blau, 1964; Cropanzano and Mitchell, 2005), this study constructs a mediational model (see Figure 1) to examine the negative association between negative workplace gossip and organizational citizenship behavior among Chinese kindergarten teachers. We argue that interpersonal trust is the key mediating mechanism. Specifically, negative gossip is associated with violations of reciprocal exchange norms and also is characterized as a negative social cue; both processes are negatively linked to teachers' trust in their colleagues (Cropanzano and Mitchell, 2005), and this association is correlated with reduced their willingness to engage in discretionary prosocial behaviors. By extending these theories to the context of Chinese kindergartens—a setting reliant on high collegial interdependence—this research is focused on clarifying a critical psycho-social process and is intended to provide practical insights for improving workplace dynamics in early childhood education.

## Methods

### Participants and procedure

Data were gathered using a questionnaire survey, a reliable approach for systematically measuring subjective constructs among kindergarten teachers via standardized survey items. This approach provides a solid empirical basis for large-scale multivariate relationship analyses and subsequent theoretical model validation (Dillman et al., 2014). The research protocol received ethical approval from the Ethics Committee of Quzhou College of Technology (Approval No.: 2025011314) and was implemented in strict compliance with the ethical guidelines set forth in the Declaration of Helsinki (World Medical Association, 2013), ensuring the protection of participants' rights to informed consent, privacy, and voluntary participation. Adopting a cross-sectional study design and convenience sampling strategy, the present investigation distributed self-report questionnaires to active kindergarten teachers across China between April 11, 2025, and May 12, 2025. The survey focused on three core constructs: negative workplace gossip, interpersonal trust, and organizational citizenship behavior. The electronic questionnaire was developed on Wenjuanxing (a leading online survey platform in China) and included the Perception of Negative Workplace Gossip Scale, Interpersonal Trust Scale, Organizational Citizenship Behavior Scale, and key demographic variables. For participant recruitment, the questionnaire link was distributed to collaborating kindergarten principals and teachers via mainstream Chinese social platforms (WeChat, DingTalk, and QQ), who then forwarded it to their workplace or administrative groups with an emphasis on voluntary participation. A standalone informed consent statement was included on the opening page of the survey, which includes the study purpose, content, and participants' rights; only those who clicked “Agree to participate” (considered as having signed a written informed consent form) were granted access to the formal survey. Prior to data collection, an *a priori* power calculation was conducted with G^*^Power 3.1.9.7 (Developed by the Heinrich Heine University Düsseldorf, Düsseldorf, Germany), with the goal of determining the minimum sample size needed for the intended multiple regression analysis (Faul et al., 2007). This calculation was configured for a multiple regression framework featuring two predictors, a small effect size (*f*^2^ = 0.075), a statistical power of 0.95, and a significance level of 0.05. Findings showed that a minimum of 209 participants was required to satisfy the predefined statistical standards. A total of 1,531 kindergarten teachers voluntarily participated and completed the questionnaire. With the goal of maintaining data quality, responses were screened to identify and remove patterned answering (e.g., consecutive identical choices) and abnormal response times (< 3 or >12 min), which are indicative of inattentive participation. Ultimately, 1,423 valid responses were retained for subsequent statistical analysis, with an effective response rate of 92.95%. The sample's detailed demographic profile is presented in detail in Table 1.

**Table 1:** Sample characteristics (*n* = 1,423).

**Variable**	**Categorical variable coding**	**Categorical variable**	**Frequency (*n*)**	**Percentage (%)**
Gender	1	Male	38	2.7
	2	Female	1,385	97.3
Marital status	1	Non-married	569	40.0
	2	Married	854	60.0
Age cohort	1	18–20	81	5.7
	2	21–30	699	49.1
	3	31–40	398	28.0
	4	41–50	202	14.2
	5	51+	43	3.0
Monthly income	1	≤ 3,000 CNY	390	27.4
	2	3,001–4,000 CNY	570	40.1
	3	4,001–5,000 CNY	262	18.4
	4	≥5,001 CNY	201	14.1

### Measures

#### Negative workplace gossip

To evaluate individuals' personal perceptions of negative workplace gossip, this research employed the Perceived Negative Workplace Gossip Scale developed by Chandra and Robinson (2009). This three-item tool measures how frequently employees feel they are the target of harmful gossip at work, with a sample item being: “Over the past 6 months, colleagues or supervisors have spread harmful information about me in the workplace.” Participants responded to each item using a 5-point Likert scale, where scores ranged from 1 (indicating *strong disagreement*) to 5 (indicating *strong agreement*). An average composite score was estimated for each member of the sample, where higher scores indicate a higher perceived level of exposure to negative workplace gossip. Prior research has provided evidence for the reliability and validity of this measurement tool within Chinese organizational contexts (e.g., Feng et al., 2025; Zhou et al., 2021), with findings linked to its cross-cultural applicability. In the present study, the scale exhibited strong internal consistency, as evidenced by a Cronbach's alpha value of 0.942.

#### Organizational citizenship behavior

Organizational citizenship behavior was measured using the Organizational Citizenship Behavior Scale, adapted for early childhood education settings. The scale was grounded in the extra-role behavior framework proposed by Podsakoff et al. (1997) and further revised by Bachrach et al. (2007). The scale comprises 10 items, addressing two primary dimensions: helping behavior: (questions 1–7) and civic virtue behaviors (questions 8–10). One illustrative item reads: “I will assist colleagues who face challenges in their work.” Participants responded to questions using a seven-point Likert scale, where 1 denoted “*strongly disagree*” and 7 denoted “*strongly agree*.” Average composite scores were calculated for each dimension, with higher scores reflecting greater engagement in organizational citizenship behaviors. In the original cross-cultural investigation by Bachrach et al. (2007), the Cronbach's alpha values for helping behavior and civic virtue were 0.81 and 0.73, respectively, with these values linked to adequate internal consistency. A prior study using Chinese samples has also provided evidence of satisfactory reliability for this tool (Zhu and Yang, 2025). In the current investigation, the scale demonstrated excellent internal consistency, with a Cronbach's alpha coefficient of 0.917.

#### Interpersonal trust

Interpersonal trust was evaluated using the Interpersonal Trust Scale, adapted for workplace environments and based on the foundational trust model constructed by Cook and Wall (1980). This assessment tool consists of seven items, with a sample statement being: “I believe the help I give to my colleagues will be returned in the future.” Participants reported their level of agreement with each item on a Likert scale (1 = *strongly disagree*, 7 = *strongly agree*), where higher scores are positively correlated with greater endorsement of trust-related beliefs. No reverse scoring was needed for the items, as all were phrased tobe consistent with positive perceptions of trust. Average composite scores were calculated, with higher values showing an association with stronger interpersonal trust among colleagues. The validity of this scale in Chinese contexts has been supported by evidence from Lu et al. (2006). This measurement tool demonstrated adequate internal consistency, with a Cronbach's alpha value of 0.885.

#### Control variables

Consistent with existing organizational behavior scholarship, demographic factors (age, gender, marital status, and monthly income) were included as control variables to address potential confounding influences—consistent with their theoretical and empirical connections to the core study constructs. Specifically, there is a higher likelihood of male or younger kindergarten teachers being targets of negative workplace gossip, while female or older counterparts demonstrate an association with greater psychological safety (Feng et al., 2025); gender is inversely linked to organizational citizenship behavior (Im and Chung, 2018), monthly income is positively correlated with it (Ko, 2008), and there is a positive association between marital status (married) and higher levels of this behavior compared to unmarried peers (Mahnaz et al., 2013); additionally, among adolescent populations, interpersonal trust exhibits a negative relationship with age (Wang et al., 2022).

### Statistical analysis

A combination of IBM SPSS Statistics 29.0 (Developed by IBM Corp., Armonk, NY, USA) and Amos 31.0 (Developed by IBM Corp., Armonk, NY, USA) was employed for statistical analyses, following a step-by-step protocol consistent with standard organizational behavior research practices. First, common method variance (CMV), a potential concern in self-report survey data, was assessed using a single-factor confirmatory factor analysis (CFA) technique (Harris and Mossholder, 1996), which is consistent with the study's primary structural equation modeling (SEM) analytical architecture. All items corresponding to the core constructs (negative workplace gossip, interpersonal trust, organizational citizenship behavior) were constrained to load on a single latent factor in Amos 31.0; a poor fit between this single-factor model and the dataset would indicate that CMV did not have a meaningful impact on the validity of the study's results (Podsakoff et al., 2012; Hair et al., 2009). Subsequently, descriptive statistics (means, standard deviations), Pearson correlations, and analysis of variance (ANOVA) were conducted to characterize variables, examine construct associations, and explore demographic differences. Next, confirmatory factor analysis (CFA) was performed via Amos 31.0 (within the SEM framework) to validate the measurement model by assessing construct reliability [Cronbach's α, composite reliability (CR)] and validity (convergent validity via AVE; discriminant validity via Fornell-Larcker criterion; Hair et al., 2009). Finally, the hypothesized partial mediation model (interpersonal trust mediating the negative workplace gossip-organizational citizenship behavior relationship) was tested using SPSS PROCESS macro [Model 4, Version 4.1 (Developed by Andrew F. Hayes, The Ohio State University, Columbus, OH, USA); Hayes, 2013]. Reliability was enhanced via 5,000 bias-corrected bootstrap resamples to compute 95% confidence intervals (CIs); effects were significant if 95% CIs excluded zero (Hayes and Preacher, 2014). Demographic factors (age, gender, marital status, monthly income) served as control variables to mitigate confounding influences.

## Results

### Common method variance and multi-collinearity testing

To assess common method variance (CMV), we conducted a single-factor confirmatory factor analysis (CFA) based on the procedure proposed by Harris and Mossholder (1996) within the structural equation modeling (SEM) framework. The single-factor model was tested using Amos 31.0, and the fit indices were poor (χ^2^/df = 45.213, RMSEA = 0.176, CFI = 0.643, TLI = 0.600). These results indicated that CMV did not significantly threaten the validity of the present study. Concurrently, SPSS 29.0 was used to test for multicollinearity among key variables (negative workplace gossip, interpersonal trust, etc.). The variance inflation factor (VIF) values ranged from 1.000 to 1.149—all well below the conventional threshold of 5. Collinearity diagnostics also revealed a maximum condition index of 15.198 (below the critical value of 25; Hair et al., 2011). These results indicated no substantial linear redundancy among variables, indicating that data quality is sufficient to support further analyses and no multicollinearity adjustments are required.

### Preliminary analyses

Table 2 included within this section shows the one-way multivariate ANOVA results for 1,423 kindergarten teachers. The mean score for negative workplace gossip was 1.85 ± 0.97 (scale 1–5), with 80.3% of the teachers obtaining a score below the 3.00 midpoint that represents the theoretical median of the scale, suggesting the reported level of negative workplace gossip was relatively low within the teaching staff of Chinese kindergartens. ANOVA results revealed significant demographic differences in negative workplace gossip scores: age (*F* = 4.44, *p* < 0.01), gender (*t* = 4.24, *p* < 0.001), and marital status (*t* = 1.65, *p* < 0.05), while no significant difference was observed for monthly income (*F* = 1.97, *p* > 0.05). More precisely, ratings of negative workplace gossip were elevated within early-career teachers (21–30 years) relative to their senior peers, male educators in comparison to female educators, and unmarried staff members vs. those who were married, while monthly income was not associated with negative workplace gossip levels. For organizational citizenship behavior, the mean score was 6.14 ± 0.87 (scale 1–7). ANOVA results demonstrated significant demographic differences in organizational citizenship behavior scores across age (*F* = 40.67, *p* < 0.001), gender (*t* = 4.41, *p* < 0.01), marital status (*t* = 21.39, *p* < 0.001), and monthly income (*F* = 2.78, *p* < 0.05). Specifically, organizational citizenship behavior scores were notably higher among older teachers (51+ years: 6.66), female teachers (6.16), married teachers (6.34), and those in the 4001–5000 CNY income bracket (6.25), relative to younger, male, unmarried, and lower-income teachers. The average score of interpersonal trust was 5.72 ± 1.05 (range from 1 to 7), with 92.3% of teachers scoring above the theoretical median (4.00), suggesting a relatively positive trust climate in kindergartens. Significant differences in interpersonal trust scores were found across age (*F* = 18.03, *p* < 0.001), marital status (*t* = 2.87, *p* < 0.001), and monthly income (*F* = 2.89, *p* < 0.05), whereas gender showed no significant association (*t* = 0.84, *p* > 0.05). Specifically, interpersonal trust scores were higher among older teachers (41–50 years: 5.98), married teachers (5.90), and those earning 4001–5000 CNY (5.83), compared to younger, unmarried, and lower-income groups.

**Table 2:** One-way multivariate analysis of variance results (*n* = 1,423).

**Sample attributes**	**Negative workplace gossip**		**Organizational citizenship behavior**		**Interpersonal trust**	
	**M (SD)**	***F/t***	**M (SD)**	***F/t***	**M (SD)**	***F/t***
**Age**						
18–20	1.74 (0.91)	4.44^**^	5.92 (1.06)	40.67^***^	5.53 (1.05)	18.03^***^
21–30	1.96 (0.98)		5.89 (0.88)		5.51 (1.08)	
31–40	1.78 (0.95)		6.43 (0.72)		5.97 (1.00)	
41–50	1.73 (0.97)		6.44 (0.79)		5.98 (0.91)	
51+	1.60 (0.82)		6.66 (0.45)		5.97 (0.89)	
**Gender**						
Male	2.48 (1.17)	4.44^***^	5.70 (1.15)	4.41^**^	5.45 (1.15)	0.84
Female	1.84 (0.96)		6.16 (0.86)		5.73 (1.05)	
**Marital status**						
Unmarried	1.92 (0.95)	1.65^*^	5.85 (0.92)	21.39^***^	5.44 (1.07)	2.87^***^
Married	1.81 (0.98)		6.34 (0.78)		5.90 (1.00)	
**Monthly income**						
≤ 3,000 CNY	1.77 (0.91)	1.97	6.05 (0.90)	2.78^*^	5.60 (1.05)	2.89^*^
3,001–4,000 CNY	1.90 (0.99)		6.15 (0.89)		5.74 (1.06)	
4,001–5,000 CNY	1.81 (0.95)		6.25 (0.78)		5.83 (1.04)	
≥5,001 CNY	1.94 (1.02)		6.18 (0.85)		5.75 (1.06)	

*M*, mean; SD, standard deviation. ^*^p < 0.05, ^**^p < 0.01, and ^***^p < 0.001.

### Measurement model validation and discriminant validity

To examine the discriminant validity of the latent constructs (negative workplace gossip, organizational citizenship behavior, and interpersonal trust), confirmatory factor analysis (CFA) was performed using Amos 31.0, with three nested measurement models compared (Table 3). The one-factor model—where negative workplace gossip, organizational citizenship behavior, and interpersonal trust were constrained to load on a single latent factor—exhibited poor fit across all indices (χ^2^ = 7686.206, χ^2^/df = 45.213, GFI = 0.605, NFI = 0.638, IFI = 0.643, TLI = 0.600, CFI = 0.643, RMSEA = 0.176). This aligns with the expectation that merging conceptually distinct constructs results in suboptimal model fit (Kline, 2015). The two-factor model—combining negative workplace gossip and organizational citizenship behavior into one factor, with interpersonal trust as a separate factor—showed modest improvement but still failed to meet standard fit thresholds (χ^2^ = 5624.597, χ^2^/df = 33.282, GFI = 0.722, NFI = 0.735, IFI = 0.741, TLI = 0.708, CFI = 0.741, RMSEA = 0.151).

**Table 3:** Fit statistics for alternative hypothesized structural equation models.

**Model**	***χ^2^***	***χ^2^/*df**	**GFI**	**NFI**	**IFI**	**TLI**	**CFI**	**RMSEA**
One-factor model	7,686.206	45.213	0.605	0.638	0.643	0.600	0.643	0.176
**Combining NWG, OCB and IT**								
Two-factor model	5,624.597	33.282	0.722	0.735	0.741	0.708	0.741	0.151
**Combining NWG and OCB**								
Three-factor model	312.563	2.562	0.979	0.985	0.991	0.986	0.991	0.033
NWG, OCB and IT								

NWG, negative workplace gossip; OCB, organizational citizenship behavior; IT, interpersonal trust.

By contrast, the three-factor model—treating negative workplace gossip, organizational citizenship behavior, and interpersonal trust as distinct latent variables—showed excellent fit to the dataset, meeting widely accepted standards (e.g., CFI ≥ 0.90, RMSEA ≤ 0.08; Ferreira-Valente et al., 2016): χ^2^ = 312.563, χ^2^/df = 2.562 (a ratio < 3 indicates well-fitting models; Kline, 2015), GFI = 0.979, NFI = 0.985, IFI = 0.991, TLI = 0.986, CFI = 0.991, and RMSEA = 0.033. These results confirm strong discriminant validity among the three constructs, supporting their conceptual independence and justifying their inclusion as distinct variables in subsequent analyses.

As presented in Table 4, reliability and validity evaluations for the measurement scales produced favorable outcomes. Composite reliability (CR) values for the construct were 0.944 (negative workplace gossip), 0.932 (organizational citizenship behavior), and 0.894 (interpersonal trust)—all above the 0.7 benchmark (Hair et al., 2009), demonstrating strong internal consistency among scale items. For convergent validity, average variance extracted (AVE) values for this factor reached 0.848 (negative workplace gossip), 0.580 (organizational citizenship behavior), and 0.554 (interpersonal trust), which met the 0.5 minimum standard (Hair et al., 2009). This suggests that each construct sufficiently captured the variance of its respective items.

**Table 4:** The correlation results between the measured variables (*n* = 1,423).

**Variables**	**α**	**CR**	**AVE**	**1**	**2**	**3**	**4**	**5**	**6**	**7**
1 Age				1						
2 Gender				−0.00	1					
3 Marital status				0.62^***^	0.00	1				
4 Monthly income				0.23^***^	−0.07^**^	0.17^***^	1			
5 Negative workplace gossip	0.942	0.944	0.848	−0.08^**^	−0.11^***^	−0.06^*^	0.04	(**0.921**)		
6 Organizational citizenship behavior	0.917	0.932	0.580	0.28^***^	0.09^***^	0.28^***^	0.06^*^	−0.30^***^	(**0.762**)	
7 Interpersonal trust	0.885	0.894	0.554	0.19^***^	0.04	0.22^***^	0.05^*^	−0.36^***^	0.68^***^	(**0.744**)
*M* (SD)				2.60 (0.91)	1.97 (0.16)	1.60 (0.49)	2.19 (0.99)	1.85 (0.97)	6.14 (0.87)	5.72 (1.05)

Diagonal entries in bold represent the square roots of average variance extracted (AVE) values. *M*, mean; SD, standard deviation. ^*^p < 0.05, ^**^p < 0.01, and ^***^p < 0.001.

For discriminant validity, the square roots of the AVE values (shown as bold diagonal entries in Table 4: 0.921 for negative workplace gossip, 0.762 for organizational citizenship behavior, 0.744 for interpersonal trust) exceeded all correlation coefficients between the focal construct and other constructs in the model. This finding satisfies the Fornell-Larcker criterion (Fornell and Larcker, 1981), confirming that each construct is empirically distinct from the others.

Table 4 also reports descriptive statistics (means and standard deviations) and the associated bivariate observed correlations among variables (*n* = 1,423). In line with theoretical predictions, negative workplace gossip was negatively correlated with both organizational citizenship behavior (*r* = −0.30, *p* < 0.001) and interpersonal trust (*r* = −0.36, *p* < 0.001), while organizational citizenship behavior and interpersonal trust showed a marked positive linear correlation (*r* = 0.68, *p* < 0.001).

### Testing the mediation model

To test the hypothesized mediation model—where interpersonal trust acts as a mediator between negative workplace gossip and organizational citizenship behavior—Model 4 of the PROCESS macro (Hayes, 2013) was used, with demographic factors (age, gender, marital status, monthly income) included as control variables. Before adding the mediator, negative workplace gossip was found to be negatively associated with organizational citizenship behavior (β = −0.24, *p* < 0.001). As reported in Table 5, when interpersonal trust was entered into the model, negative workplace gossip showed a significant negative relationship with interpersonal trust [β = −0.38, *p* < 0.001, 95% CI (−0.43, −0.33)]. In turn, interpersonal trust was positively associated with organizational citizenship behavior [β = 0.51, *p* < 0.001, 95% CI (0.48, 0.55)]. Additionally, negative workplace gossip retained a direct negative association of statistical significance with organizational citizenship behavior [β = −0.05, *p* < 0.01, 95% CI (−0.09, −0.02)]. Although statistically significant, the magnitude of this direct effect was relatively small.

**Table 5:** Interpersonal trust as a mediator linking negative workplace gossip to organizational citizenship behavior.

**Predictors**	**Interpersonal trust**			**Organizational citizenship behavior**		
	β	**SE**	**95% CI**	β	**SE**	**95% CI**
Constant	5.56^***^	0.34	[4.89, 6.23]	2.27^***^	0.24	[1.80, 2.74]
Age	0.06	0.04	[−0.01, 0.13]	0.12^***^	0.02	[0.08, 0.17]
Gender	0.04	0.16	[−0.27, 0.35]	0.28^**^	0.10	[0.08, 0.48]
Marital status	0.33^***^	0.07	[0.20, 0.46]	0.10^*^	0.04	[0.02, 0.19]
Monthly income	0.04	0.03	[−0.01, 0.09]	−0.01	0.02	[−0.04, 0.03]
Negative workplace gossip	−0.38^***^	0.03	[−0.43, −0.33]	−0.05^**^	0.02	[−0.09, −0.02]
Interpersonal trust				0.51^***^	0.02	[0.48, 0.55]
*R^2^*	0.17			0.50		
*F*	58.46^***^			232.97^***^		

The total number of bootstrap samples was 5,000. ^*^p < 0.05, ^**^p < 0.01, ^***^p < 0.001.

**Figure 2:**
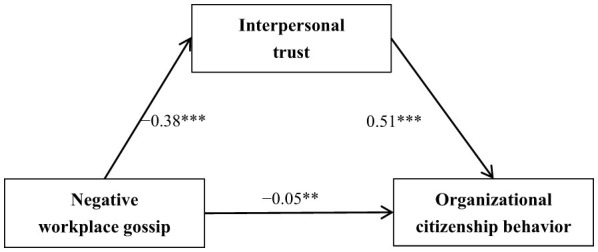
Mediation pathways of negative workplace gossip, organizational citizenship behavior, interpersonal trust. **p < 0.01 and ***p < 0.001.

To examine the indirect relationship between negative workplace gossip and organizational citizenship behavior, a bootstrapping procedure with 5,000 resamples was used. The findings showed that interpersonal trust significantly mediated the association between negative workplace gossip and organizational citizenship behavior [β = −0.19, *p* < 0.001, 95% CI (−0.23, −0.16)]. Specifically, the 95% bootstrapped confidence interval (based on 5,000 samples) did not include zero, statistically suggesting the presence of a mediation effect. Further analysis revealed that interpersonal trust functioned as a partial mediator, with the indirect effect accounting for 79.17% of the total effect. Figure 2 depicts the mediation pathway with its respective calculated standardized path coefficients.

## Discussion

Drawing on social exchange theory, this investigation explored the relationship between negative workplace gossip and organizational citizenship behavior among Chinese kindergarten teachers, while testing the potential mediating role of interpersonal trust. This study advances existing research and extends the practical application of social exchange theory by situating it within the distinct professional and cultural context of early childhood education in China.

Hypothesis 1 predicted a negative association between negative workplace gossip and organizational citizenship behavior within the sample of Chinese kindergarten teachers, an assertion derived from social exchange theory and supported by recent empirical research (Martinescu et al., 2021). Social exchange theory argues that the reciprocal nature of interactions—both between individuals and their organizations and among coworkers—is related to employee behavioral responses (Blau, 1964). From this perspective, employees tend to respond to positive organizational and interpersonal interactions with voluntary behaviors that foster collective workplace wellbeing, such as organizational citizenship behavior. Conversely, negative workplace gossip is associated with weaker conditions for positive social exchange and is linked to lower levels of employees' willingness to engage in organizational citizenship behavior (Martinescu et al., 2021).

A prior three-wave time-lagged study has further corroborated the adverse effects of negative workplace gossip on organizational citizenship behavior. Xie et al. (2022) found that employees targeted by negative workplace gossip exhibit significantly lower commitment to organizational citizenship behavior. Their findings suggest that negative workplace gossip erodes the organizational identification of targeted individuals, and this diminished identification directly reduces their motivation to engage in proactive, organizationally beneficial behaviors. This mechanism is particularly evident in early childhood education, as teamwork and collaborative interactions—such as jointly developing curriculum plans, helping each other in child care, and jointly organizing educational activities—are essential for ensuring service quality (Bautista et al., 2023). Negative workplace gossip among kindergarten teachers is associated with reduced trust and cooperation among colleagues, and such trust and cooperation are linked to the occurrence of these collaborative behaviors, which in turn correlates with reluctance to invest additional time and effort in organizational citizenship behavior (Danish, 2024). To further strengthen the support for Hypothesis 1, additional empirical evidence directly linking negative workplace gossip to organizational citizenship behavior in educational contexts is supplemented. Akkaya (2019) found an association between negative workplace gossip and reduced voluntary helping behaviors among primary school teachers—a core dimension of organizational citizenship behavior—with a hostile interpersonal climate potentially acting as a relevant factor, which is consistent with the findings of this study. Kaya (2015) further confirmed link between negative workplace gossip and diminished civic virtue and altruistic behaviors among employees (key components of organizational citizenship behavior), and this associative conclusion is further supported in the context of kindergarten teachers in our study. Li et al. (2021) conducted a meta-analysis and found that negative workplace gossip was significantly and negatively related to targets' positive behaviors, including organizational citizenship behavior. This provides general support for Hypothesis 1.

Hypothesis 2 proposes that interpersonal trust acts as a mediator in the relationship between negative workplace gossip and organizational citizenship behavior, an assertion consistent with social exchange theory and supported by growing empirical evidence. Social exchange theory emphasizes that trust serves as the cornerstone of sustainable positive social exchange relationships (Blau, 1964). Employees are only willing to make long-term, voluntary contributions like organizational citizenship behavior when they trust their colleagues and organization (Lee et al., 2024), as these behaviors entail a degree of vulnerability and reliance on others' reciprocity. Negative workplace gossip, as a destructive interpersonal phenomenon, is associated with lower levels of this foundational trust, with such reduced trust linked to interruptions in the positive social exchange process and correlated with lower organizational citizenship behavior. Furthermore, a study rooted in social exchange theory and conducted with 257 Chinese employees confirmed that negative workplace gossips associated with lower affective commitment (a construct closely linked to interpersonal trust), which in turn correlates with reduced organizational citizenship behavior (Cui, 2020). These results jointly support the potential mediating role of interpersonal trust outlined in Hypothesis 2, highlighting that negative workplace gossips associated with impaired trust—a factor linked to the willingness to engage in organizational citizenship behavior among Chinese kindergarten teachers.

The hypothetical relationships discussed above exhibit particular relevance to the occupational context of Chinese kindergarten teachers, owing to the unique characteristics of their work environment. Kindergarten teachers operate in high-emotion, high-interaction settings where the quality of interpersonal relationships is associated with service delivery and child development outcomes (Sottimano et al., 2017). Unlike many other professions, their work requires constant collaboration and mutual reliance (Stasio et al., 2020)—for example, coordinating daily routines, addressing children's unexpected needs, and implementing educational programs collectively. As such, interpersonal trust is not merely a facilitating factor but a fundamental requirement for effective job performance. Negative workplace gossip in this context is therefore likely to be particularly consequential, as it is associated with challenges to the trust-based relationships necessary for collaboration. Moreover, Chinese cultural values of harmony and collectivism (Bautista et al., 2023) are associated with more pronounced psychological consequences of negative workplace gossip: being targeted is related to deviations from group harmony norms, and is associated with greater feelings of exclusion and lower willingness to participate in collective organizational citizenship behavior. This cultural and professional context aligns with a stronger negative association between negative workplace gossip and organizational citizenship behavior, as well as the mediating function of interpersonal trust, making Hypotheses 1 and 2 especially relevant to the study's sample.

### Theoretical contributions

From the perspective of empirical context expansion, this study expands the scope of negative workplace gossip research by focusing on Chinese kindergarten teachers—a professional group with unique occupational traits (e.g., high emotional labor, frequent interpersonal interactions, and heavy reliance on teamwork)—thus challenging the overrepresentation of corporate employees in existing literature. Most prior studies have centered on employees in enterprises and institutions, examining how negative workplace gossip relates to attitudes (Hassona et al., 2022) and work engagement (Cheng X. et al., 2023). In contrast, the unique occupational traits of kindergarten teachers make the transmission and effects of negative workplace gossip distinct from those in corporate settings. By exploring the relationship between negative workplace gossip and organizational citizenship behavior among Chinese kindergarten teachers, this study extends negative workplace gossip research into the under-explored field of early childhood education, offering a new empirical basis to examine cross-contextual variations in the effects of negative workplace gossip.

Second, this study advances understanding of the mediating mechanisms linking negative workplace gossip to organizational citizenship behavior by introducing interpersonal trust as a mediator. Prior research has identified multiple mediating pathways, including psychological safety (Xie et al., 2022) and self-esteem rooted in identification with the organization (Song and Guo, 2022). Nevertheless, limited research has explored the mediating function of interpersonal trust, particularly within early childhood education settings. Interpersonal trust is the cornerstone of interpersonal interactions in organizations, and its formation and maintenance are particularly important in kindergartens where collaborative work is frequent. This study demonstrates an association between negative workplace gossip, reduced interpersonal trust (among kindergarten teachers' trust in colleagues and the organization), and lower willingness to engage in organizational citizenship behavior. This finding not only adds to the existing body of knowledge on mediating mechanisms between negative workplace gossip and organizational citizenship behavior, but also offers a novel theoretical lens to explain the association between negative workplace gossip and prosocial behaviors in educational institutions.

From the perspective of theoretical application extension, this study extends the practical application of social exchange theory to the field of early childhood education by revealing the theoretical mechanism underlying the relationship between negative workplace gossip and organizational citizenship behavior in this specific context. Social exchange theory posits that organizational interactions are rooted in the expectation of mutual benefit, and trust serves as a critical prerequisite for social exchange to occur (Blau, 1964; Cropanzano et al., 2017). Existing research has applied social exchange theory to explain associations between leadership behavior, organizational support, and organizational citizenship behavior in corporate settings (e.g., Elshaer et al., 2022; Mandiyasa et al., 2022), but few studies have applied this theory to interpret the impact of negative workplace gossip in early childhood education—where interpersonal trust is even more critical due to its direct association with children's development. Grounded in social exchange theory, this study argues that negative workplace gossip undermines the interpersonal trust foundation essential for social exchange among kindergarten teachers, which in turn reduces their willingness to expend extra effort on organizational citizenship behavior. By verifying this associative chain, this study not only extends social exchange theory's application to early childhood education but also provides fresh empirical evidence for its explanatory value in educational management, addressing the call from Cropanzano et al. (2017) to examine social exchange theory's applicability in more specialized organizational contexts.

### Implications for practice

Drawing on the findings of this study—that negative workplace gossip is associated with lower organizational citizenship behavior among kindergarten teachers, both directly and indirectly through an association with reduced interpersonal trust—we propose practical implications uniquely tailored to the kindergarten environment, which is characterized by integrated care and education, frequent *in-situ* collaboration, and close peer interactions in daily child care. These recommendations are designed to be actionable, aligned with the core work of kindergarten teachers, and targeted at mitigating the adverse effects of gossip, safeguarding interpersonal trust, and promoting organizational citizenship behavior.

First, design early childhood education-specific targeted support for kindergarten teachers to buffer against gossip's impacts, integrated with daily care and education work. Given that kindergarten teachers rely heavily on peer collaboration in daily child care (e.g., morning check-ins, nap time supervision, and after-class summary), we recommend establishing “Mentor-Mentee Co-Care Partnerships” that pair novice and experienced teachers not only for professional guidance but also for joint responsibility in daily child care tasks. For example, paired teachers can co-lead a morning activity theme (e.g., “animal friends”) or jointly manage nap time for a class, fostering *in-situ* trust and a sense of belonging through collaborative care practices (Doan, 2013; Park and Choi, 2025). Additionally, implement “Emotional Check-In Circles” before daily morning meetings—a brief, informal 5-min ritual where teachers share one positive or challenging moment related to child care (e.g., “a child actively helped a peer today” or “I felt stressed when coordinating parent communication yesterday”). This ritual provides emotional support tailored to the unique stressors of early childhood education work and reduces the emotional vulnerability that is linked to greater susceptibility to gossip among teachers (Moe-Byrne et al., 2022).

Second, implement kindergarten-contextualized gossip prevention strategies embedded in daily work routines to preserve interpersonal trust. Instead of generic “explicit codes of conduct,” we propose integrating “no-gossip” principles into staff training on child care ethics, framing gossip as a threat to collaborative care—for example, how gossip disrupts consistent child guidance between teachers (e.g., conflicting rules for children's behavior due to mistrust caused by gossip). To address information asymmetry (a key antecedent of gossip), establish “Daily Care Briefings” (10-min daily sessions) where teachers share real-time updates on children's needs (e.g., a child's dietary restriction), classroom events (e.g., a sudden change in the afternoon activity), and administrative arrangements, eliminating the “information vacuum” that fuels gossip (Himmetoglu et al., 2018). Furthermore, foster open communication through “Curriculum Co-Creation Workshops” (biweekly 30-min sessions) where teachers collaboratively design theme-based child care and education activities (e.g., a seasonal parent-child activity or a cross-class art project), turning formal collaboration into opportunities for direct, respectful peer interaction that discourages gossip propagation (Cislaghi et al., 2019).

Third, develop early childhood education-aligned trust-building initiatives closely linked to the core work of kindergarten teachers—child care and curriculum implementation. Replace generic “trust-building initiatives” with practice-embedded activities: (1) Launch “Collaborative Care Challenge Tasks,” such as planning and executing a large-scale parent-child sports day or a cross-class thematic education event (e.g., “autumn harvest exploration”). In these tasks, teachers rely on each other's expertise in child development, activity design, and parent communication to achieve shared goals, which is associated with strengthened mutual competence and benevolence trust (Farah, 2023). (2) Establish a “Peer Recognition for Prosocial Care Behaviors” system tailored to early childhood education, where teachers nominate colleagues for organizational citizenship behavior actions specific to child care—such as “staying late to support a colleague whose child was sick and couldn't pick up their own child” or “adapting a lesson plan to support a peer's teaching style for a shy child.” Recognize these behaviors in monthly “Star Teacher” ceremonies focused on “collaborative care excellence,” aligning rewards with the core values of early childhood education and reinforcing positive interpersonal interactions (Ennida and Allouani, 2023; Martinescu et al., 2021). This not only relates to greater trust but also is associated with sustained organizational citizenship behavior by validating the prosocial actions that are essential to high-quality kindergarten education.

### Limitations and future directions

This research has several limitations that merit recognition and highlight promising avenues for future investigation. First, its cross-sectional design prevents definitive causal conclusions about the relationships between negative workplace gossip, interpersonal trust, and organizational citizenship behavior. Longitudinal or experimental designs would more clearly establish whether trust mediates the impact of gossip on organizational citizenship behavior. Second, the use of a convenience sample of Chinese kindergarten teachers may restrict the generalizability of the results. Notably, the sample had an extremely high female proportion (97.3%)—reflecting the actual demographic but leading to extremely low statistical power for gender-based analyses (e.g., *t*-tests in Table 2) due to only 38 male participants, making these comparisons statistically unreliable. Additionally, cultural norms surrounding gossip, trust, and prosocial behavior in collectivist, education-oriented contexts may differ markedly from those in individualistic or non-educational workplaces. Future studies should employ random sampling across diverse occupational and cultural settings to enhance external validity. Third, all variables were measured using self-report instruments, which introduces potential issues of common method bias and socially desirable responding. Incorporating multi-source data—such as peer nominations of organizational citizenship behavior or supervisor ratings of trust—could provide more objective and triangulated evidence. Finally, while interpersonal trust was examined as a mediator, other psychological or contextual factors (e.g., emotional intelligence, perceived organizational support, or moral identity) may also shape how employees respond to negative gossip. Investigating these variables could yield richer theoretical insights and more nuanced interventions for fostering positive workplace behaviors among early childhood educators.

## Conclusions

Drawing on social exchange theory, this investigation examines the association between negative workplace gossip and organizational citizenship behavior among Chinese kindergarten teachers, with interpersonal trust as a proposed mediator. Key findings demonstrate that negative workplace gossip is associated with reduced interpersonal trust among kindergarten teachers, which in turn is linked to lower engagement in organizational citizenship behavior. Theoretically, this study advances negative workplace gossip research by extending its scope to early childhood education, deepens insight into the mediating mechanisms linking the focal variables, and expands the application of social exchange theory to educational management contexts. Practically, the findings provide targeted implications for kindergartens: prioritize support for high-risk groups (younger, male, and unmarried teachers), prevent negative workplace gossip through explicit norms and transparent communication, and cultivate interpersonal trust via collaborative activities and incentive systems. These measures are associated with mitigating the adverse impact of gossip, safeguarding interpersonal trust, and encouraging organizational citizenship behavior, thereby relating to improved operational effectiveness of early childhood education institutions.

## Data Availability

The raw data supporting the conclusions of this article will be made available by the authors, without undue reservation.
